# Therapeutic advances in the topical treatment of cutaneous leishmaniasis: A review

**DOI:** 10.1371/journal.pntd.0009099

**Published:** 2021-03-03

**Authors:** Marium Azim, Saeed Ahmad Khan, Saleem Ullah, Shafiq Ullah, Syed Ishtiaq Anjum

**Affiliations:** 1 Department of Pharmacy, Institute of Chemical and Pharmaceutical Sciences, Kohat University of Science and Technology, Kohat, Khyber Pakhtunkhwa, Pakistan; 2 Department of Zoology, Kohat University of Science and Technology, Kohat, Khyber Pakhtunkhwa, Pakistan; Centro de Pesquisa Gonçalo Moniz-FIOCRUZ/BA, BRAZIL

## Abstract

Cutaneous leishmaniasis has been endemic since decades. Millions of cases are reported worldwide specially in developing and underdeveloped countries. There are 2 major types of cutaneous leishmaniasis based on the causating species found in different regions of the world. These include New and Old World cutaneous leishmaniasis, which are self-healing, but if not treated, these may cause severe scars and many other complications like mucosal involvement. The conventional gold standard treatment for both types is mainly intralesional or parenteral administration of antimonial. Lately, a great deal of research has been done on development of topical treatment based on single agent or combination therapy. This review summarizes the current state of literature regarding therapeutic outcome of topical treatment against cutaneous leishmaniasis caused by different species in different regions.

## Introduction

Leishmania is an intracellular parasite from trypanosomatidae family among protozoa; consisting of almost 20 species which are transmitted by female sandflies (90 species), causing leishmaniasis. There are 3 main types of leishmaniasis; visceral or kala-azar leishmaniasis (VL), cutaneous leishmaniasis (CL), and mucocutaneous leishmaniasis. VL is the deadliest form, while CL the most common form. Throughout the world, about 1.5 to 2 million new cases occur per year, among which 500,000 cases consist of VL and 1 to 1.5 million cases of CL. In 2018, a report showed that over 85% of new CL cases has occurred in Afghanistan, Algeria, Bolivia, Brazil, Colombia, Iran, Iraq, Pakistan, the Syrian Arab Republic, and Tunisia [1,2].

CL produces skin lesions that develop in weeks to months after a female sandflies bite, leaving behind permanent scars and serious disability if untreated [2]. CL is further classified into 2 subtypes, i.e., New World cutaneous leishmaniasis (NWCL) and Old World cutaneous leishmaniasis (OWCL). NWCL is mainly caused by *Leishmania braziliensis* and *Leishmania mexicana* complex, which are spread in Western Hemisphere, while OWCL is caused by *Leishmania donovani*, *Leishmania major*, *Leishmania infantum*, *Leishmania aethiopica*, and *Leishmania tropica* complex, which are endemic to Eastern Hemisphere [3,4].

Leishmaniasis was previously considered a diverse group of syndromes because of many parasite species involved and exhibiting clinical manifestations. On the basis of this diversity, different alternative treatments have long been investigated for therapeutic effectiveness. Conventionally, parenteral administration of sodium stibogluconate (SSG) and meglumine antimonate (MA) has been the most common approach [5,6]. However, due to the high systemic toxicity of injectable and oral drugs, topical treatment options have been studied, indicating a promising treatment alternative [5].

In this review, topical treatment strategies have been discussed in detail along with their outcomes to highlight all the available options and advancements made for future developments.

## Methods

We have searched the literature related to topical treatment and/or studied the treatment of simple cutaneous leishmaniasis (SCL) including OWCL and NWCL. Literature review included reports from 1990 till the time this review has been written (May 2020), which include clinical as well as animal studies done for drug candidates with proven outcomes. Online databases PubMed, NLM Medline, and Google Scholar were used to retrieve all the articles. The articles written and published in English language were included. Moreover, the primary focus was on the articles mentioning topical formulations of antileishmanial drugs, and articles reporting significant therapeutic outcome and showing potential antileishmanial activity in the treatment of simple CL. The terms cutaneous leishmaniasis, OWCL, NWCL, topical treatment, paromomycin, gentamicin, urea, methyl benzalkonium chloride, miltefosine, liposomal miltefosine, amphotericin B, liposomal amphotericin B, Buparvaquone, and imiquimod were used alone and in combination during searching different databases. Other terms searched were morphine, rifampicin, artemether AND cutaneous leishmaniasis, antifungal drugs AND topical treatment AND cutaneous leishmanisis, gold standard treatment regimens AND sodium stibogluconate AND topical formulations. Any other relevant literature was retrieved and was also reviewed when relevant with updated data.

## Findings

Three major areas have been found worth discussing while reviewing the literature about topical treatment of simple cutaneous leishmaniasis including:

- the conventional treatments alone and in combination;
- the difference in responses to the topical formulations by the type of CL, i.e., the species responsible for OWCL and NWCL; and
- new dosage forms of conventional drugs and new drugs under research.

## Topical treatment strategies in clinical trials

### Topical products containing single drug

#### Paromomycin ointment

Paromomycin (PA) is an aminoglycosides antibiotic. It was previously used for gastrointestinal infections such as amoebiasis and giardiasis. In 1960s, it was reported to have antileishmanial effects in CL [7]. PA was initially investigated in different combinations with other drugs such as methyl benzalkonium chloride (MBCl), urea, and gentamicin (another aminoglycoside) [8–12].

In a double blind randomized controlled trial (RCT) in Iran, Asilian and colleagues reported clinical outcome of duration-based therapy with 15% Paromomycin (PA) ointment (by Pharmacia Upjohn) among OWCL single lesioned uncomplicated CL patients caused by *L*. *major*. They reported 59% and 74% cure rate (CR) after 2 weeks treatment and 4 weeks of treatment, respectively. Moreover, after 45 days of treatment, both groups of patients needed rescue treatment with parenteral treatment. The frequency of rescue treatment was reported to be high (33%) in patients treated for 2 weeks as compared to that of (19%) patients treated for 4 weeks. However, in the long-term follow-up (day 105), there was no significant difference in the clinical outcome of 4-week treatment versus 2-week treatment. The study concluded that the cost of treatment could be reduced up to 1 USD using 15% PA ointment. Furthermore, it could also prevent 75% patients (with uncomplicated CL caused by *L*. *major*) from exposure to the highly toxic parenteral treatment [13].

In a recent study, Soto and colleagues (2019) have reported a positive outcome by the change of drug delivery system (DDS) used for the topical application of PA in leishmaniasis caused by *L*. *brazilensis* (NWCL). A total of 15% PA ointment was formulated in a hydrophilic vehicle previously reported for good adsorption properties. For comparison, intralesional (IL) Pentamidine injection (Sanofi-Aventis, Bogota, Columbia) administered on days 1, 3, and 5 at the dose of 120 μg/mm^2^ at lesion site. The PA-hydrophilic formulation showed intent-to-treat (ITT) cure rate of 77.5% with a better tolerance, as compared with the positive control with an ITT cure rate of 70%. These results suggest that delivery system can greatly affect penetration and in turn therapeutic outcome of topical treatment. However, further investigation is needed on different formulation against other species causing CL (Table 1) [14].

**Table 1:** Paromomycin and combination regimens therapeutic outcome in OWCL and NWCL.

Drug regimen	OWCL			NWCL		
	Manufacturer/Brand	Outcome and regimen	Reference	Manufacturer/Brand	Outcome and regimen	Reference
Paromomycin (PA) ointment	Pharmacia Upjohn	74% CC (4 weeks), 59% CC (2 weeks). *(Species*: *L*. *major*, Uncomplicated CL)	[13]	Paromomycin (Asia Foresight-care group Limited Xian, Shaanxi Province, China)	ITT-CR of 77.5% with the combination DDS ointment (OD for 20 days), ITT-CR of 10% with the negative control aquiphilic vehicle, and ITT-CR of 70% with the positive control pentamidine injection (IL at the dose of 120 μg/mm^2^ on days 1.3 and 5). *(Species*: *L*. *brazilensis*)	[14]
				PA-hydrophilic ointment (Study site laboratory)		
				Pentamidine injection (Sanofi-Aventis, Bogota Columbia)		
12% PA with 12% MBCl ointment	Farmitalia Carlo Erba and Warner Lambert, Italy	Complete eradication after treatment for 10 to 20 days with both compositions in comparison to WSP (Placebo) *(Species*: *L*. *major)*	[31]	-	-	-
12% PA with 5% MBCl ointment						
12% PA with 12% MBCl ointment	Humatin (PA only)	Poor response (35.7%) with twice daily application for 15 days compared to zero response with oral KTZ dosing for 30 days (400 mg in adults and 200 mg in peads). *(Species*: *Not mentioned)*	[32]	-	-	-
	(Nizoral tablets)					
15% PA with 12% MBCl ointment	Research laboratory of Isfahan Pharmacy College, Isfahan, Iran	Poor response (41.2%) compared to PDT (93.4%) *(Species*: *L*. *major)*	[33]	Pharmacy of university at Heidelberg, Germany	Approx. same (high) responses (72%, 90%, and 85%) to both regimens of OD for 20 days and BD for 10 days at follow-ups. *(Species*: *L*. *panamenisis)*	[34]
	-	-	-	TEVA pharmaceutical industries (ointment)	CR of 58% with the ointment (BD for 10 days), 53% with ointment followed by 7 days of 20 mg/kg/day MA administration, 84% with ointment followed by 3 days of 20 mg/kg/day MA administration (MA, *the gold standard*), and 20% with MA administration alone for 20 days. *(Species*: *L*. *braziliansis panamensis)*	[36]
				Injection MA (Specia, Bogota, Colombia)		
	-	-	-	TEVA pharmaceutical industries (ointment)	Overall CR of 54% with the ointment in antimony naïve patients, 2.17 times greater effect than placebo (WSP) and more cost effective than the *gold standard* MA treatment. *(Species*: *L*. *braziliansis*, *75% and L*. *mexicana*, *25%)*	[37]
15% PA and 5% MBCl ointment	-	-	-	Sodium Stibogluconate, SSG (Pentostam)	Enhanced CR of 90% when followed by 7 days of SSG regimen (*the gold standard)* at 20 mg/kg/day and CR of 42% when followed by 3 days of SSG regimen at 20 mg/kg/day as compared to treatment with either drug alone. *(Species*: *L*. *braziliansis panamensis)*	[35]
15% PA and 10% urea ointment	Farmitalia Carlo Erba (Pharmacis Upjhon) Milan, Italy	No significant therapeutic outcome reported after BD application for 2 weeks. *(species*: *L*. *major*, *L*. *tropica*, *and L*. *papatasi)*	[38]	-	TR of 40% by 15% PA/10% urea ointment (BD for 30 days) was obtained as compared to 48.3% TR obtained by 15% PA/12% MBCl ointment (for 30 days) and 80.6% TR by *gold standard* IM administration of MA (20 mg/kg/day for 10 days). Lowest relapse reported with urea combination (10.5%) *(Species*: *not mentioned)*	[44]
	10% Urea (Eucerin, Isfahan Pharmacy College and Research Laboratory)	Failed outcome of 16.6% recovery and 72.9% failure after BD application of the ointment for 45 days as compared to 41.7% recovery and 3.7% failure by IL administration of *the gold standard* MA at 1.5 g/5 ml/week dose for 12 weeks max. *(species*: *L*. *major and L*. *tropica*	[39]	-	Inadequate response among children after application of the ointment TDS for 4 weeks	[42]
	Injection MA (Glucantime)					
	15% PA (Razak Foundation Tehran) and 10% urea (Eucerin, Beiersdorf, Hamburg, Germany)	CCR of 17% in treatment and placebo group each with a failure rate of 57% and 51%. *(Species*: *not mentioned)*	[40]	-	-	-
	15% PA (Razak Foundation Tehran)	CR at 1-week interval for ointment was 68% (0.5 mg/mm^2^/d BD for 20 days) and ID administration of MA was 66% (every other day for 20 days) No significant difference among ointment and the *gold standard* therapeutic outcome. *(Species*: *L*. *major)*	[41]	-	-	-
	Injection MA (Rhons-Poulenic Rorer, Paris, France)					
15% PA and 0.5% Gentamicin	Paromomycin (Farmatalia) Gentamicin (Schering) WR279396	CCR of 94% (at day 50) and no relapse (at day 180) was obtained with the ointment as compared to the placebo CCR of 74%. *(Species*: *L*. *major)*	[45]	-	CR of 61% with the combination ointment (BD for 20 days) with a reduction in duration of cure up to 35 days compared to 56 days with placebo. *(Species*: *L*. *pamnamensis)*	[47]
	WR279396 (Teva Pharmaceuticals)	Equivalent CCR of 81% by the combination ointment and 82% by the 15% PA only ointment (OD for 20 days) was reported as compared to 58% CCR with the vehicle. *(Species*: *not mentioned)*	[46]	-	CR of 87% with the combination ointment (BD for 20 days) as compared to CR of 60% with 15% PA ointment after 6 months of follow-up. *(Species*: *L*. *pamnamensis)*	[48]
	-	-	-	-	Efficacy reported 79% with the combination ointment and an equivalent efficacy with 15% PA ointment only (OD for 20 days) *(Specie*: *Not mentioned)*	[49]
	-	-	-	(WR279396) 0.5% gentamicin and 15% PA ointment	Equivalent CCR reported with both types of occlusion methods with the combination ointment (OD for 20 days), i.e., CCR of 91.7% with gauze-tape and 79.2% with polyurethane occlusion. *(Species*: *Not mentioned)*	[50]

BD, bis in die (2 times a day); CC, clinical cure; CCR, clinical cure rate; CL, cutaneous leishmaniasis; DDS, drug delivery system; IL, intralesional; IM, intramuscular; ITT-CR, intent-to-treat cure rate; KTZ, ketoconazole; MA, meglumine antimonite; MBCl, methyl benzalkonium chloride; NWCL, New World cutaneous leishmaniasis; OD, omne in die (once a day); OWCL, Old World cutaneous leishmaniasis; PA, paromomycin; PDT, photodynamic therapy; SSG, sodium stibogluconate; TDS, transdermal delivery system; TR, therapeutic rate; WSP, white soft paraffin.

#### Imiquimod cream

Imiquimod (IMQ) is a member from the class of drugs known as imidazo-quinolines with the ability to modulate the immune response by up-regulating different inflammatory mediators, i.e., cytokines like tumor necrosis factor alpha (TNF alpha), interleukin 12 (IL-12), interferon alpha (IFN alpha), and interferon beta (IFN beta) [15,17]. Previously, due to the immunomodulatory action, IMQ was used for its antiviral actions and anti-inflammatory actions for the treatment of skin cancers [16–18]. However, recently, IMQ has been found to help in vanishing the CL lesions both in vitro and in vivo, by inducing strong TH1 immune responses [18] and generation of nitric oxide in the macrophages [15]. Over the past decade, a number of studies have been conducted to study its clinical efficacy of IMQ against CL. For instance, in 2001, a group of 12 MA-resistant Peruvian patients suffering with NWCL were effectively treated by combination of topical IMQ 5% cream and IM administration of MA. Topical IMQ seemingly overcame the resistance; as a result, CR of 90% was achieved by topical application of 5% IMQ cream for 20 days on every alternate day and IM injection of MA at the dose of 20 mg/kg/day for 20 days [19]. Similarly, a comparative study was performed for IMQ 7.5% cream (for 20 days on alternate day alone and in combination with IV-administered MA (20 mg/kg/day for 20 days). The CR after 1-month follow-up was 17% and 57% for IMQ cream alone and IV-administered MA alone, respectively, while the combination of 7.5% IMQ cream and IV-administered MA showed CR of 100% after 1-month follow-up. An effective and better cosmetic outcome was achieved with the combination treatment; moreover, least relapse rate (0% relapse at 3 months of follow-up) was observed with combination treatment as compared to IMQ 7.5% cream (100% relapse at 3 months of follow-up) and MA injection (43% relapse at 3 months of follow-up) alone [20]. Furthermore, in 2005 and 2009, during double blind RCTs of the combined therapy (systemic MA injection and topical IMQ 5% cream) among Peruvian patients confirmed that it improved the quality of scar caused in patients with NWCL. Consequently, therefore, it was considered the first line treatment in Peru for CL patients [21,22].

Contrarily, Khalili and colleagues [23] have found that topical treatment with IMQ 5% cream did not show any significant improvement for Syrian patients with OWCL (common species, *L*. *tropica*). Ali Raza and colleagues also determined that the response of systemically administered MA (20 mg/kg/day) was not affected by topical application of IMQ 5% cream in OWCL caused by *L*. *tropica* [24]. Thus, it can be concluded that topical IMQ against OWCL is not as promising as observed in NWCL, and further studies need to be performed in countries where OWCL species are commonly reported (Table 2) [25].

**Table 2:** Imiquimod and Amphotericin B regimens therapeutic outcome in OWCL and NWCL.

Drug regimen	OWCL			NWCL		
	Manufacturer	Outcome and regimen	Reference	Manufacturer	Outcome and regimen	Reference
IMQ 5% cream	-	No significant effect *Species*: *L*. *tropica*	[25]	IMQ 5% cream (Aldara by 3M Pharmaceuticals, St. Paul, Minnesota)	Effective outcome with a CR of 90% with a combination regimen of the cream on alternate days (20 days) and IM injection of MA at the dose of 20 mg/kg/day (20 days) *Species*: *L*. *peruviana*	[19]
				MA injection (Glucantime by Aventis Pasteur)	Improvement in scars with combination regimen. *Species*: *L*.*(Viannia) barzilliensis* and *L*.*(Viannia) peruviana*	[21,22]
	-	No significant improvement when combined with regimen of systemic MA injection (20 mg/kg/day) *Species*: *L*. *tropica*	[24]			
IMQ 7.5% cream	-	-	-	-	Effective outcome with combination regimen of cream on alternate days (20 days) and IV injection of MA at the dose of 20 mg/kg/day (20 days) As compared to either treatment alone. *Species*: *Not mentioned*	[20]
**Amphotericin B**						
Liposomal AmB formulation	Injection MA (Glucantime)	No significant difference in efficacy with topical regimen (BD for 8 weeks) in comparison to IL Inj. MA (once a week). *Species*: *L*. *major and L*. *tropica*	[26]	-	-	-
3% AmB cream	-	-	-	Anfoleish	Not significant improvement, 39.4% CR with BID regimen and 35.3% CR with TID regimen (for 4 weeks). *Species*: *L*. *panamensis*, *L*. *braziliensis*, and *L*. *guyanensis*	[30]

AmB, Amphotericin B; BD,bis in die (2 times a day); CR, cure rate; IL, intralesional; IMQ, imiquimod; MA, meglumine antimonite; NWCL, New World cutaneous leishmaniasis; OWCL, Old World cutaneous leishmaniasis.

#### Amphotericin B lipid formulation

Amphotericin B (AmB) is a polyene macrolide antibiotic; previously isolated from *Streptomyces nodoses* has been reported to have antileishmanial activity in addition to its well-known antifungal properties [26–28]. Since long AmB topical administration has been investigated for the cure of CL to prevent its systemic toxicities because of its highly nephrotoxic nature [28]. Vardy and colleagues pioneered investigating the effectiveness of ethanol-based AmB-lipid formulation in patients with CL caused by *L*. *major* species for topical treatment which extends up to weeks or even months [29]. In Iran, a comparative study was performed on OWCL patients. The treatment included intralesional MA (Glucantime) injection (1 to 2 ml once a week for 8 weeks) in comparison with topical liposomal AmB formulation (250 μg/ml, 3 to 7 drops twice daily into each lesion, for 8 weeks). No significant difference was observed in efficacy, i.e., 48.3% ITT was seen in MA-treated group compared to 44% ITT in liposomal AmB-treated group [26]. Similarly, no promising results were observed in Colombian patients with uncomplicated NWCL. For instance, Lopez and colleagues studied oil in water (o/w) emulsion containing 3% AmB (Anfoleish) cream in patients with NWCL caused by *L*. *panamensis*, *L*. *braziliensis*, and *L*. *guyanensis* in Colombia. CR of 39.4% and 35.3% was observed for BID and TID regimens, respectively. Thus, it can be concluded that topical AmB is neither effective in OWCL nor in NWCL. Nevertheless, further studies need to be performed based on novel formulations that might improve results in clinical practice (Table 2) [30].

### Topical product containing combination of drugs

#### Paromomycin/methyl bezalkonium chloride (MBCl) combination ointment

Different response in clinical outcomes was reported when PA was used in combination with other drugs. For instance, complete eradication of OWCL caused by *L*. *major* was achieved with ointment containing 15% PA and 12%/5% MBCl. Variation in MBCl concentration showed no significant differences in clinical outcome [31]. On the other hand, when 15% PA and 12% MBCl ointment was compared with oral therapy of ketoconazole tablets in Turkish patients with OWCL, a poor response was observed at the end of 4 weeks posttreatment. For instance, CR 35.7% was reported after application of topical treatment twice daily for 15 days. Whereas, no cure was achieved with once daily oral dosing of ketoconazole tablets 400 mg (adults) and 200 mg (children) for 30 days [32]. Similarly, photodynamic therapy (PDT) was compared with ointment containing 15% PA with 12% MBCl in OWCL caused by *L*. *major*. It was found that PDT was more effective (93.4%) as compared to ointment (41.2%), when treated twice daily for 4 weeks [33]. However, against NWCL caused by *L*. *panamenisis*, PDT and ointment showed no difference in therapeutic outcome (72% at the end of 50 days) [34]. Moreover, the healing rates for NWCL cases were also low compared to that of OWCL cases [31].

In another comparative study, PA and MBCl ointment (15% and 5%, respectively) was comparted with SSG and MA during their Phase II and Phase III trials. Initially, the impact of treatment was studied in 2 cohorts of NWCL caused by *L*. *braziliansis panamensis*. Both the groups received topical ointment 15% PA and 5% MBCl twice daily for 10 days. However, one of the groups was then followed by 7 days or 3 days of SSG administration parenterally at the dose of 20 mg/kg/day. The CR reported was 90% in group 1 (treated for 7 days) and 42% in group 2 (treated for 3 days), and the overall therapeutic outcome of the combination treatment with both drugs during treatment and after follow-ups at 1.5, 3, 6, 9, and 12 months posttreatment showed an enhanced effect as compared to either of the drug used alone [35].

In continuation during Phase III partial RCT in Colombia, Soto and colleagues studied the effect of PA MBCl ointment against NWCL caused by *L*. *braziliansis panamensis*. First group received 15% PA and 12% MBCl ointment twice a day for 10 days, and it showed 58% CR. Patients in second group were treated with topical treatment, followed by intramuscular (IM) injection of MA at the dose of 20 mg/kg/day for 7 days showed 53% CR. Third group showed topical treatment followed by 3 days of MA injection of similar dose IM, and it showed 84% CR. Moreover, fourth group received MA injection for 20 days, and it showed poor response of 20% CR. These differences among Phase II and Phase III trials depict that there might be many reasons for variation in CR. For instance, the formulation was provided by a pharmaceutical industry which prepared it under good manufacturing practices (GMP) during Phase III trial as compared to Phase II trial formulation, where the formulation was extemporaneously prepared. Moreover, during Phase III trial, the improved assessment provided clarification of results [36]. In another report, an overall CR of 54% was achieved with 15% PA and 12% MBCl ointment, which was 2.17 times greater outcome than placebo group. The clinical outcome was 90% CR, which was in good agreement with the previously studied gold standard MA treatment (at the dose of 20 mg/kg for 10 to 20 days) in antimony-naïve patients diagnosed with *L*. *braziliensis* (75%) and *L*. *mexicana* (25%). This trial also suggested a cost-effective treatment option with an expenditure of 6 USD (in case the ointment was locally prepared) compared to 280 USD expenditure of parenteral MA administration (Table 1) [37].

#### Paromomycin and urea combination ointment

The combination topical treatment with 15% PA and 10% urea hadn’t proved to be effective in many RCTs, majority of which were performed in Iran. Asilian and colleagues performed a study on Iranian patients with OWCL caused by *L*. *major* and in some cases by *L*. *tropica*. Treatment done was twice daily application of 15% PA and 10% urea for 2 weeks. Follow-up after the end of treatment showed no significant therapeutic outcome [38]. Similarly, in another study, 15% PA and 10% urea formulation (manufactured by Beiresdorf, Hamburg, Germany) has also been reported as ineffective for OWCL. Moreover, 15% PA and 10% urea formulation studied against Leishmaniasis caused by *L*. *major* and *L*. *tropica* showed only 16.6% complete recovery and 72.9% failure when applied twice daily for a long duration of 45 days. In comparison, the IL administration of MA (Glucantime) at 1.5 g/5 ml weekly for 12 weeks proved to be effective (41.7% complete recovery and 3.7% failure) [39]. In another RCT in Iran, during follow-up at 30 days obtained, investigating 15% PA and 10% urea formulation, 17% complete CR was observed and 57% failures were reported [40]. In another study, topical 15% PA and 10% urea ointment was compared with intradermal (ID) injections of MA; the ointment was applied twice a day for 20 days at the dose of 0.5 mg/mm^2^/d and was compared with ID injection of MA alternate days for 20 days among confirmed *L*. *major* patients in an endemic area. The CRs after 1-week treatment showed no significant differences, i.e., 68% and 66% for ointment and ID injections, respectively. No difference in clinical outcome was observed after followed up of 6 months [41]. Ponce and colleagues reported almost 0 cured cases in children with NWCL caused by *L*. *mexicana* and *L*. *chagasi* in Honduras when treated with similar 15% PA and 10% urea ointment, with slightly modified regimen of application of 3 times a day for 4 weeks [42]. Thus, it can be concluded that 15% PA and 10% urea ointment has no substantial beneficial effects in terms of treatment for both OWCL and NWCL cases.

In a double blind RCT among Ecuadorian patients, 15% PA and 12% MBCl ointment twice daily and 15% PA and 10% urea ointment, applied for 30 days each, were evaluated comparatively with the gold standard treatment of IM injection of MA for treating NWCL caused by *L*. *braziliensis*, *L*. *panamensis*, *L*. *guyanensis*, *L*. *Mexicana*, *and L*. *amazonensis* [43]. Approximately equivalent therapeutic responses were obtained for both topical formulations. After 6 weeks of follow-up, 15% PA and 12% of MBCl ointment group showed 48.3% CR, while 15% PA and 10% urea group showed 40% CR. Moreover, the response was 79.3% and 70% after 12 weeks of treatment, respectively. Administration of IM injection of MA 20 mg/kg/day for 10 days showed CR of 80.6% after 6 weeks and 91.7% after 12 weeks. Relapse rate reported after initial healing was lowest for PA and urea ointment (9.5%) as compared to PA and MBCl ointment (17.4%) and injection MA (15.2%). The therapeutic outcome showed injection MA to be effective, but the relapse rate and profile of side effects showed topical treatments especially PA and urea ointment more promising for future research in both NWCL and OWCL (Table 1) [44].

#### Paromomycin and gentamicin combination ointment

The topical combination 15% PA and 0.5% gentamicin (WR279396) in a hydrophilic base is one of the widely studied formulations for the treatment of CL caused by various species including both OWCL and NWCL. These formulations showed promising results during different phases of clinical trials. In 2009, Phase II double blind RCT by Salah and colleagues reported the safety and effectiveness of 15% PA and 0.5% gentamicin ointment in Tunisian patients with OWCL caused by *L*. *major*. CR was observed to be 94% on day 50 follow-up as compared to CR of 74% placebo group. Moreover, no relapse was seen at day 180 after treatment completion [45]. In 2013 during a Phase III comparative RCT, 15% PA ointment alone and in combination with 0.5% gentamicin were studied. The treatment was done once a day for 20 days. Similar outcome was reported in both treatment groups, i.e., 81% CR for 15% PA ointment group and 82% CR for 15% PA and 0.5% gentamicin [46]. The results are in good agreement with the previous reports for effectiveness of PA ointment against OWCL [13].

An effective response was reported for PA and gentamicin combination ointment during Phase II trial among Colombian patients with *L*. *panamensis* which caused NWCL. The duration of cure was reduced to 35 days with 61% CR after twice-a-day dosing for 20 days, as compared to CR of 55% and 56 days with placebo [47]. Moreover, in Panama where majority of patients have *L*. *panamensis-*induced NWCL, the PA and gentamicin ointment showed equivalent responses to that of 15% PA ointment [48,49]. During Phase II trials, the PA and gentamicin ointment showed 87% improvement as compared to 15% PA ointment (showing 60% improvement) at follow-up after 6 months when treated twice a day for 20 days, and almost an equivalent percentage of CR was observed for both regimens [48]. While in Phase III trial the efficacy reported was 79% and 78% for 15% PA and 0.5% gentamicin ointment-treated group and 15% PA ointment-treated group, respectively, when applied once daily for 20 days [49]. The results show an equivalent efficacy of the combination formulation in both OWCL and NWCL.

Salah and colleagues also studied the effect of 2 types of occlusion methods on the efficacy of 0.5% gentamicin and 15% PA (WR279396) ointment when applied once daily for 20 days in Tunisian patients with a confirmed CL. Gauze-tape and polyurethane occlusion methods have been tested which did not report significant differences in CCR after 20 days. Responses of 91.7% and 79.2% were obtained for gauze-tape and polyurethane occlusion, respectively (Table 1) [50].

## Topical treatment strategies in preclinical stages

### Buparvaquone gel

Buparvaquone (BPQ) is hydroxyl naphthoquinone studied for having antiprotozoal activity since long [51]. Its antileishmanial activity was first studied in vitro by Croft and colleagues [52]. BPQ itself is a poor water-soluble moiety and, therefore, its pro-drugs are formulated to improve topical drug delivery. A water-soluble pro-drug of BPQ, 3-Phosphono-oxymethyl-buparvaquone (3-POM-BPQ), was found to be the most potent molecule among the investigated derivatives [53]. Several formulations of BPQ and 3-POM-BPQ were developed to enhance the penetration and were tested on full thickness BALB/c skin as well as human skin. A hydrous gel, i.e., w/o emulsion of BPQ and an anhydrous-gel-A of 3-POM-BPQ showed greater skin penetration [54]. In vivo studies showed the efficacy of these formulations in BALB/c *L*. *major* model by decreasing parasite burden and delaying lesion progression. When early and late treatment initiation has been compared, more notable results were obtained for the group where treatment was started after 3 days of onset of infection. Common adverse effect reported was irritation. These studies showed a greater potential in BPQ and its pro-drugs to be used in future topical treatments of CL [55] in addition to novel strategies [56,57].

### Miltefosine gel

Miltefosine (MT), also known as Hexadecylphosphocholine, is a phospholipid derivative having potent anticancer and in vitro leishmanicidal activity. It is approved for oral administration in breast cancer and VL [58,59]; however, there is a need for the formulation of its topical dosage form in order to lower the resistance and toxicity among patients with CL [59]. The mechanism of MT is not understood yet; however, studies have shown that it had increased intracellular membrane fluidity on the uppermost layer of skin (stratum corneum, SC). This effect of MT on the fluidity can be exploited for the development of topical MT formulations to be used in the treatment against CL [60]. In another report, 6% w/v MT formulation was studied in vivo on female BALB/c mice infected with *L*. *major* species; it showed poor penetration of the drug with least improvement of the lesion size and parasite load along with intense skin irritations after 5 days of treatment [61]. Contrarily, another study reported 85% to 99% reduction in parasite in the lymph nodes and spleen of mice infected with *L*. *mexicana and L*. *major* CL when treated with topical 6% MT [62]. Similarly, 0.5% miltefoscine Carbopol gel reduced the lesion size by 84% to 100% with no parasite detection in BALB/c mice infected with NWCL species, i.e., *L*. *(Viannia) braziliensis* and *L*. *(Viannia) panamensis*. These discrepancy in results might be due to variation in causating species, treatment duration, and formulation components [63].

Najafian and colleagues have recently reported that nanoliposomal MT has had greater therapeutic outcome as compared to conventional formulation when studied in vitro in dermal macrophages of BALB/c mice infected with Iranian strain of *L*. *major* species [64]. Similarly, a recent study showed 4% liposomal MT formulation that needs further investigation for CL [65]. Both these studies reinforced that liposomes can enhance therapeutic outcome of MT for treatment of CL.

### Morphine cream

Morphine is an alkaloid acting as an immunomodulatory agent. It has been found to be able to improve the healing of CL wounds [66] when applied topically. The wound size was potentially reduced among BALB/c mice inoculated with the parasite *L*. *major* when morphine ointment was applied topically as compared to the placebo used [67]. This effect can be used as an adjuvant treatment in future for treatment of CL.

### Artemether PVA solution

Artemether, a free radical producing antiparasitic drug, also showed promising antileishmanial effects against various leishmania species, i.e., *L*. *major* [68,69] and *L*. *infantum* [70] both in vitro and in vivo. Artemether suspended in polyvinyl alcohol (PVA) solution showed significantly better outcome in terms of lesion size, comparted to glucantime-PVA system when applied topically BALB/c mice infected with *L*. *major* [68]. This makes artemether an interesting candidate for further studies.

### Liposomal sodium stibogluconate gel

Recently, SSG has been loaded a nanodeformable liposomal (NDL) DDS for topical application among *L*. *tropica*-infected BALB/c mice. The SSG-NDL gel was reported to greatly enhance the effect of topical formulation via significantly reducing the lesion size (4-fold increase in activity) and also reducing the IC50 value from 1.65 mg/ml of plain SSG ointment to 1.3 mg/ml of SSG-NDL gel. This report is also an example of how novel DDS can greatly enhance the therapeutic outcome of the conventionally used drugs [71].

### Ketoconazole and fluconazole cream

Azole antifungals are used for a wide range of fungal infections including yeast infections of various mucocutaneous surfaces like mouth, throat, esophagus, and genital organs. Additionally, azole antifungals have also shown antileishmanial properties in combination with pentavalent antimonial drugs. Researchers investigated 1% fluconazole cream twice daily for approximately 6 weeks in combination with once weekly IL glucantime. It was found that fluconazole cream potentiated the effect of glucantime (64% CR) and can be used in treating CL lesion [72]. Another study also showed a similar result of enhanced effect of SSG when combined with ketoconazole (NELs), BALB/c mice infected with *L*. *maxicana*. However, it needs further investigation for effective regimens to be used in the topical treatment of CL [73].

## Conclusion and future directions

In developing and third world countries, CL is still endemic because of limited number of drugs available to patients. Moreover, most of these treatment regimens require parenteral administration which poses serious discomfort for patients, poor patient compliance, higher cost of therapy, and lower therapeutic outcome among patients. Various topical treatments are being studied since long to overcome these problems since it is more convenient dosage forms for CL that provide high concentration of drug at the site of infection. This review has highlighted that variation in the *leishmania* species in different regions has different therapeutic outcome. Topical treatment combined with parenteral administration has shown higher therapeutic efficacy. Several new and old drug candidates with modified dosage forms are in pipeline which will increase topical therapeutic options for treatment of CL. In the future, pharmacodynamic and pharmacokinetic analysis of drugs and new dosage forms used in the topical treatment of CL will be discussed in detail.

**Key learning points**

- Treatment of both types of cutaneous leishmaniasis has greatly been advanced from parenteral to topical treatment options with significant therapeutic outcomes.
- Several combination treatments comprising of topical and parenteral modalities are developed and studied in different regions reporting different *Leishmania* species. Variation in therapeutic outcome has been reported for different species in different regions.
- There are many findings which showed that the modifications in the drug delivery systems from conventional to novel dosage forms can greatly enhance the therapeutic outcome of many topically used drugs; it therefore emphasizes to do more future research on improving the dosage forms to get optimum therapeutic outcome and to counter the resistance toward these agents.

**Top five papers**

1. Salah AB, Buffet PA, Morizot G, Massoud NB, Zâatour A, Alaya NB, et al. WR279, 396, a third generation aminoglycoside ointment for the treatment of Leishmania major cutaneous leishmaniasis: a phase 2, randomized, double blind, placebo controlled study. 2009;3(5):e432.
2. Ben Salah A, Ben Messaoud N, Guedri E, Zaatour A, Ben Alaya N, Bettaieb J, et al. Topical paromomycin with or without gentamicin for cutaneous leishmaniasis. N Engl J Med. 2013;368(6):524–32.
3. Arevalo I, Tulliano G, Quispe A, Spaeth G, Matlashewski G, Llanos-Cuentas A, et al. Role of imiquimod and parenteral meglumine antimoniate in the initial treatment of cutaneous leishmaniasis. 2007;44(12):1549–54.
4. Layegh P, Rajabi O, Jafari MR, Emamgholi Tabar Malekshah P, Moghiman T, Ashraf H, et al. Efficacy of Topical Liposomal Amphotericin B versus Intralesional Meglumine Antimoniate (Glucantime) in the Treatment of Cutaneous Leishmaniasis. J Parasitol Res. 2011:656523.
5. Carneiro G, Aguiar MG, Fernandes AP, Ferreira LAM. Drug delivery systems for the topical treatment of cutaneous leishmaniasis. Expert Opin Drug Deliv. 2012;9;1083–1097.
